# Candidate Gene Polymorphisms and their Association with Glycogen Content in the Pacific Oyster *Crassostrea gigas*

**DOI:** 10.1371/journal.pone.0124401

**Published:** 2015-05-07

**Authors:** Zhicai She, Li Li, Haigang Qi, Kai Song, Huayong Que, Guofan Zhang

**Affiliations:** 1 National & Local Joint Engineering Laboratory of Ecological Mariculture, Institute of Oceanology, Chinese Academy of Sciences, Qingdao, Shandong, China; 2 University of Chinese Academy of Sciences, Beijing, China; The Ohio State University, UNITED STATES

## Abstract

**Background:**

The Pacific oyster *Crassostrea gigas* is an important cultivated shellfish that is rich in nutrients. It contains high levels of glycogen, which is of high nutritional value. To investigate the genetic basis of this high glycogen content and its variation, we conducted a candidate gene association analysis using a wild population, and confirmed our results using an independent population, via targeted gene resequencing and mRNA expression analysis.

**Results:**

We validated 295 SNPs in the 90 candidate genes surveyed for association with glycogen content, 86 of were ultimately genotyped in all 144 experimental individuals from Jiaonan (JN). In addition, 732 SNPs were genotyped via targeted gene resequencing. Two SNPs (Cg_SNP_TY202 and Cg_SNP_3021) in *Cg_GD1* (glycogen debranching enzyme) and one SNP (Cg_SNP_4) in *Cg_GP1* (glycogen phosphorylase) were identified as being associated with glycogen content. The glycogen content of individuals with genotypes TT and TC in Cg_SNP_TY202 was higher than that of individuals with genotype CC. The transcript abundance of both glycogen-associated genes was differentially expressed in high glycogen content and low glycogen content individuals.

**Conclusions:**

This study identified three polymorphisms in two genes associated with oyster glycogen content, via candidate gene association analysis. The transcript abundance differences in *Cg_GD1* and *Cg_GP1* between low- and the high-glycogen content individuals suggests that it is possible that transcript regulation is mediated by variations of Cg_SNP_TY202, Cg_SNP_3021, and Cg_SNP_4. These findings will not only provide insights into the genetic basis of oyster quality, but also promote research into the molecular breeding of oysters.

## Introduction

The Pacific oyster *Crassostrea gigas* is an economically important mollusk that is widely cultivated all over the world, though China is the largest producer. Global production expands continuously at a rate of four million tons per year (FAO, 2014, http://www.fao.org). Oysters are an important source of glycogen, as it can make up 20–40% of their dry weight. The glycogen in oysters is associated with their energy metabolism and summer mortality, and can affect both gonad development and gametogenesis [1–3].

There have been many studies of glycogen metabolism in humans, and its molecular mechanism has been well addressed [4–8]. As it is an important nutrient, glycogen has been extensively studied in domesticated animals such as pigs, lambs, and chickens [9–12]. In bivalves, glycogen-related research lags far behind that conducted on humans and domesticated animals. To date, the target traits for the genetic dissection and breeding of oysters have mainly been associated with rapid growth and increased stress resistance [13–17]. Studies investigating the genetic basis of glycogen content in mollusks are scarce.

Glycogen is a branched glucose polymer present in many organisms, from bacteria and archaea to humans [18]. Glycogen metabolism is very conservative, and the number of enzymes directly involved in this process is quite small [19]. Glycogen phosphorylase has been extracted and purified from the adductor muscles of oysters and scallops by Keiko Hata [20,21], while Bacca cloned the oyster glycogen synthase gene and carried out the real-time fluorescent quantitative PCR, finding out that the expression level of glycogen synthase was consistent with seasonal changes in glycogen content [22]; however, the molecular mechanism behind glycogen metabolism is still unclear.

Oyster glycogen content is a quantitative trait, changing seasonally. Its production varies widely among different individuals [22,23]. It cannot be detected visually or in living animal, and thus conventional breeding methods are not suited to efforts aiming to breed oysters for nutritional quality. Marker-assisted selection seems to be the best alternative, and requires the screening of the genomic variation related to the target traits. Association analysis, including genome wide association study (GWAS) and candidate gene association study (CGA), is utilized extensively in order to link genomic and phenotypic variations. The pathway-driven identification and selection of candidate genes has proven to be a successful strategy for association studies [24], especially those investigating simple metabolic pathways, such as the metabolism of glycogen.

Single nucleotide polymorphisms (SNPs), mainly include the polymorphisms in a DNA sequence caused by single nucleotide mutations. SNPs have been widely used in genetic research and especially in association analysis due to the advantages of higher abundance, genotyping efficiency, data quality, genome-wide coverage and analytical simplicity [25–29]. The oyster genome and transcriptome predict abundant SNPs and genes related to the oyster glycogen metabolism [30,31], providing the genetic resources for our study. Of the various methods of SNP genotyping, high resolution melting HRM is powerful and cost-effective, and does not have the drawbacks of other methods [32–35]. In addition, the improved two-step HRM using a small amplicon is a more economic and effective method for moderate-throughput SNP genotyping in organisms with high polymorphism [31]. The cost decrease of next generation sequencing makes genotyping by sequencing (GBS) a more accessible method for association analysis in many species [36–40]. Additionally, targeted resequencing is an ideal method for candidate gene association studies. Targeted resequencing, more cost-effective and rational for sequencing target regions [41], is usually used to validate genes and mutations screened by association analysis [42,43]. The regulation of gene expression is an important genetic component of quantitative traits, so analysis of the mRNA expression of the associated genes is usually used to further validate results [24,44,45].

We herein sort out the gens related to glycogen metabolism and work out a direct and simplified pathway, from which we screen a set of candidate genes for association analysis applying HRM in a wild population. Then, we validate the results in an independent population using targeted gene resequencing. Our research aims to identify the genes and mutations associated with glycogen content, and provide genetic tools for the marker-assisted selection of oysters.

## Materials and Methods

### Ethics Statement

The oysters used in this study were marine-cultured animals, and all experiments were conducted according to the regulations established by the local and central government. No specific permissions were required to collect oysters or conduct the experiments described.

### Experimental animals

Wild oyster larvae from Jiaonan (JN, N35°44ʹ, E119°56ʹ), Qingdao, China were attached and cultured locally. At the age of 16 months, 144 individuals were sampled randomly for association analysis. The adductor muscle and mantle of each individual were dissected individually and snap-frozen in liquid nitrogen prior to being stored at -80°C for later use. Meanwhile, for the independent analysis, 144 individuals from Shentanggou (STG, N36°22ʹ, E120°42ʹ, 110 kilometers from JN), Qingdao were attached, cultured, and sampled in the same manner as those from JN. Sixty-four of the 144 STG individuals, consisting of the 32 individuals with the highest glycogen content, and the 32 with the lowest, were selected for targeted gene resequencing. In addition, gill samples from STG oysters with extreme phenotypic rankings, divided into high glycogen content (n = 16) and low glycogen content (n = 16) groups, were used for mRNA expression assays for the associated genes. Prior to dissection, shell height, length, and width, total weight, and tissue weight were measured. Details of the samples used in the experiment are shown in S1 Table.

### Glycogen content assay

The adductor muscles of oysters from both JN and STG were used for the glycogen content assay along with a kit for detecting liver and muscle glycogen content (Nanjing Jiancheng Bioengineering Institute), according to the procedure described below: First, 30–60 mg adductor muscle were removed and rinsed using physiological saline, then weighed with a 0.1 mg electronic balance after the moisture had been blotted up using filter paper. Then, the sample was added to a test tube with a volume of alkaline liquor three times the weight of the sample. Then the samples were incubated for 20 min at 100°C in a water bath, followed by cooling under running water. Then, a volume of distilled water 16-fold the sample was added to the above hydrolysate for subsequent usage. Subsequently, 2 mL of the color regent was added and the tubes were incubated in a 100°C water bath for 5 min. Finally, the OD value of each tube was measured using a spectrophotometer under light of 1 cm diameter. The blank control and standard were set for each group (S2 Table). The glycogen content was calculated according to the formula below:

Glycogen content (mg/g) = (OD of test group/OD of standard group) ∙ 0.01 ∙ 20 ∙ 10/1.11

where 1.11 is the coefficient of glucose content detected using this method, converted to glycogen content.

### Screening of candidate genes and SNPs genotyping

We summarized the glycogen metabolism pathway using established knowledge, and searched for the associated enzyme-encoding genes in the predicted oyster gene set [30]. All examined genes encoding important enzymes were assigned as candidate genes related with glycogen content, and each of the candidate genes was aligned using blastn and blastp from NCBI (http://blast.ncbi.nlm.nih.gov/Blast.cgi) for sequence confirmation. As important mutations associated with the target traits are more likely located in the key genes and their surrounding regulatory regions [38,42,43], we studied not only the candidate gene regions, but also their upstream and downstream regions. Specifically, SNP validation using HRM and association analysis were performed for all the candidate genes in JN to locate the associated genes and SNPs. The SNPs were further validated by HRM in STG population. In addition, some selected candidate genes in the STG population were resequenced via Illumina sequencing to acquire more trait-associated SNPs.

#### HRM analysis

An improved small-amplicon HRM analysis [31] was used for SNP validation and genotyping. The genomic DNA of each individual was extracted from the mantle using the E.Z.N.A SQ Tissue DNA Kit. For each SNP predicted by the genome and transcriptome sequences of the candidate genes [30,31], we designed specific primers using the Primer Premier 5.0 software. The amplified size was limited to 50–100 bp, and only one SNP was included. Primers producing a single clear band on the polyacrylamide gel were used for the HRM analysis with eight samples. After amplification, LC Green and an interior label were added to each sample, followed by denaturation at 94°C for 10 min. Samples were then placed in a Light-Scanner 96 device (Idaho Technology, Salt Lake City, UT, USA) for HRM analysis. Genotypes were identified using Light Scanner 96 software (Idaho Technology), according to their melting temperatures. All the validated SNPs were genotyped in the JN population, while the associated SNPs were genotyped in STG population using the HRM analysis method described above.

#### Targeted gene resequencing

The regions targeted for sequencing included the selected genes and their 3 kb upstream and downstream regions. In addition to genes associated with glycogen content at association analysis stage I (details in the materials and methods section “Association and other statistical analysis”), *Cg_GS* and *Cg_GP*, enzymes known to rate-limit the process of glycogen metabolism, were selected as target genes for resequencing.

Prior to resequencing, cloning and sequencing were performed to fill the gaps in the genome sequence and ensure that the PCR amplification was specific to the target region. The filled genome sequence then function as the reference genome for subsequent SNP calling. The targeted regions were amplified using several overlapping pairs of primers designed using primer premier 5.0 software. All amplicons were linked to T vectors, and then transformed into the competent *E*. *coli* cells. After one night of incubation, monoclones were picked for sequencing via the Sanger sequencing method. Finally, 42 pairs of primers were selected for use in amplification of the target genes.

For each of the 64 (S1 Table) samples from STG, all PCR products were detected via agarose gel electrophoresis, then equally mixed. Then, the mixture of each individual was fragmented randomly and barcoded, in order to identify individuals after sequencing. Finally, the 64 samples were divided into four groups, each containing 16 individuals. We constructed libraries for each group and sequenced them on Illumina HiSeq 2000 in BGI.

Adapter sequences and contaminated sequences were removed, and reads in which low-quality (Q ≤ 5) bases accounted for more than half of the read were filtered out. Then, we detected SNPs for each sample using SOAPsnp (http://soap.genomics.org.cn/soapsnp.html), after aligning the reads to the reference sequence using SOAP2 (http://soap.genomics.org.cn/soapaligner.html) software. The SNP calling was made following the filtering criteria below: base quality higher than 20, sequencing depth higher than five and lower than 200, unique read alignment, missing rate lower than 10%, and a minor allele frequency higher than 0.05.

### Association and other statistical analysis

SPSS version 16.0 was used to conduct the descriptive statistical analysis of the glycogen content. We also performed a correlation analysis to determine whether glycogen content was correlated with shell height, shell length, shell width, total weight, or tissue weight. Glycogen content among different genotypes was compared via ANOVA, using SPSS version 16.0.

We performed the first-stage candidate gene association analysis using the genotyped SNPs from JN to screen for associated loci and genes. Significantly associated loci were further validated in the STG population via HRM analysis. In addition, we carried out the second-stage association analysis with SNPs obtained via resequencing the associated genes from the STG population. We performed this analysis using GAPIT version 2.08 software via a mixed linear model approach implemented in EMMA, taking into account population structure and relative kinship [46–48]. The statistical model was:

Y=Xβ+Zu+e

where Y is the phenotype glycogen content, β is an unknown vector containing fixed effects such as the genetic marker, population structure (Q), and the intercept u, an unknown vector of random additive genetic effects. X and Z are the known design matrices, and e is the unobserved vector of the random residual.

We used PHASE version 2.1.1 to predict the SNP haplotype of the associated genes in the 64 resequenced samples from STG, then analyzed the association between haplotype and glycogen content with SPSS version 16.0, using one-way ANOVA.

### The mRNA expression analysis of associated genes

The transcript abundances of the associated genes was detected using real-time quantitative PCR (Q-PCR). Total RNA was isolated from the gills of each individual in both the high glycogen content and the low glycogen content groups, using TRIzol reagent (Invitrogen). The integrity and concentration was detected by agarose gel electrophoresis and Nanodrop 2000 spectrophotometer (Thermo), respectively. First-strand cDNA was synthesized using PrimeScript RT reagent Kit with gDNA Eraser (TaKaRa), following the manufacturer’s instructions. Quantitative Real-Time PCR was performed using an ABI 7500 FAST Real-time PCR System. The elongation factor (EF) gene was used as a normalizer gene. The amplification was carried out in a reaction volume of 20 μL, containing 10 μL of 2X SYBR Green PCR Master Mix (ABI), 0.4 μL each of the forward and reverse primers (10 μM), 1 μg of template cDNA, and ddH_2_O for a final volume of 20 μL. The cycling program was as follows: 50°C for 2 min, 95°C for 2 m, then 40 cycles of 95°C for 3 s and 60°C for 30 s. The comparative CT method was used to determine fold-changes in gene expression, calculated as 2^-ΔΔCT^.

## Results and Discussion

### Phenotypic statistics

The glycogen content of the experimental oysters ranged from 2.1 to 10.4 mg/g, with a mean ± std. value of 5.3 ± 1.7. The degree of variation was more than five-fold. Glycogen content was normally distributed (P > 0.05) (S1 Fig). The correlation analysis showed that glycogen content was not correlated with shell height, shell length, shell width, total weight, or tissue weight. This indicates that glycogen content is a quantitative trait suitable for association analysis.

### Candidate gene screening of the glycogen metabolism pathway

The glycogen metabolism pathway is summarized in S2 Fig. Ninety genes encoding eight important enzymes were ultimately selected as candidate genes (S3 Table). They were classified into three groups: 1) glycogen anabolism enzyme, including glycogen synthase (*Cg_GS*, one encoding gene), phosphoglucomutase (*Cg_PG*, four encoding genes), and HK hexokinase (*Cg_HKH*, three encoding genes); 2) glycogen catabolism, including glycogen phosphorylase (*Cg_GP*, two encoding genes), glycogen debranching enzyme (*Cg_GD*, two encoding genes), and glycosyltransferase (*Cg_GT*, 74 encoding genes); 3) glycogen metabolism regulators, including glycogen phosphorylase kinase (*Cg_GPK*, three encoding genes) and protein phosphatase (*Cg_PP*, one encoding gene).

### Genotyping by HRM and resequencing

#### High resolution melting (HRM) analysis

We designed a total of 677 pairs of primers in total for the 90 candidate genes (Table 1). Two hundred and ninety-five SNPs were validated preliminarily (S4 Table) for eight individuals from JN, and 86 were finally genotyped for all 144 JN experimental individuals (S1 Table). The overall ratio of SNP validation for eight oysters was 43.6% (295/677), which was consistent with the results of the oyster transcriptome SNP validation, 43.9% (1301/2962) [31]. The SNPs genotyped across all 144 JN individuals only accounted for 29.2% (86/295) of the total validated SNPs. This failure of the HRM analysis was mainly due to the presence of unpredicted SNPs in the PCR amplicon [31]. Only small amplified regions with a single SNP were well-genotyped via the HRM method, as unexpected SNPs in the PCR amplicon led to the failure of the HRM analysis. The presence of the unexpected SNPs in the oyster was derived from the high polymorphism and limited number of individuals used for SNP prediction in the non-genic region.

**Table 1:** Statistics for the polymorphisms of the glycogen content candidate genes.

Enzymes	Encoding genes	Designed Primers	Validated SNPs	Genotyped SNPs
*Cg_GS*	1	22	9	5
*Cg_PG*	4	37	12	0
*Cg_HKH*	3	23	14	1
*Cg_GP*	2	42	14	1
*Cg_GD*	2	65	33	21
*Cg_GT*	74	400	216	50
*Cg_GPK*	3	76	26	6
*Cg_PP*	1	12	4	2
Total	90	677	295	86

Validated SNPs were validated by HRM using 8 samples, genotyped SNPs were genotyped by HRM using all the 144 samples. *Cg_GS*, glycogen synthase. *Cg_PG*, phosphoglucomutase. *Cg_HKH*, HK hexokinase. *Cg_GP*, glycogen phosphorylase. *Cg_GD*, glycogen debranching enzyme. *Cg_GT*, glycosyl transferase. *Cg_GPK*, glycogen phosphorylase kinase. *Cg_PP*, protein phosphatase.

#### Targeted gene resequencing

To validate the candidate SNP markers from the stage I association analysis and to get more glycogen content related SNPs, we sequenced five selected target genes (S5 Table) in the independent population of STG. The five target genes included the associated *Cg_GD1* at the stage I and four extra key genes selected basing on the prior knowledge. We designed 85 pairs of primers in total to amplify the target gens as well as the 3 kb of both upstream and downstream of them. After PCR amplification, cloning, and sequencing, 42 pairs of primers were retained while 43 pairs discarded for producing no amplification products, or non-targeted amplicons. The details of the remaining 42 pairs of primers are shown in S6 Table. Sixty-four oysters consisting of 32 high-glycogen and 32 low-glycogen content oysters from the original 144 wild individuals (S1 Table) were sequenced. The descriptive statistics of the 32 low- and the 32 high-glycogen content individuals are shown in S7 Table. The mean glycogen content of the low group was 3.73 mg/g, with a range of 4.69 mg/g—2.19 mg/g. In the high glycogen-content group, the mean glycogen content was 8.83 mg/g, while the maximum and the minimum values were 10.2 mg/g and 7.84 mg/g, respectively.

The Q20 values of all filtered 1.26G reads were greater than 97.69%, indicating that the sequencing quality was very high. By aligning the reads to the 90.6 kb reference sequence, we identified 732 high quality SNPs. Table 2 shows the distribution of the SNPs on each candidate gene. The total number on each of the sequenced genes ranged from 32 in *Cg_GD2* to 256 in *Cg_GS*. On average, one SNP calling was made per 124 bp, which was sufficient for association analysis. The read coverage rate of each individual mostly ranged from 60–70% and the mapping rate from 40–50%, which was much lower than that achieved via the method using selector probes for targeted region enrichment [41]. This might be a result of the existence of highly duplicated sequences in the oyster genome [30], and the fact that the sequenced amplicons were not the targeted ones.

**Table 2:** The distribution of SNPs for each resequenced gene.

Gene ID	Length(kb)	Total number	CDS	3'UTR	5'UTR
CGI_10016530	23.8	155	13	10	0
CGI_10023027	8.9	54	14	5	0
CGI_10005175	14.9	32	13	0	0
CGI_10005875	21	235	28	13	3
CGI_10017418	22	256	32	8	27
Total	90.6	732	100	36	30

### Candidate gene association analysis

**Fig 1:**
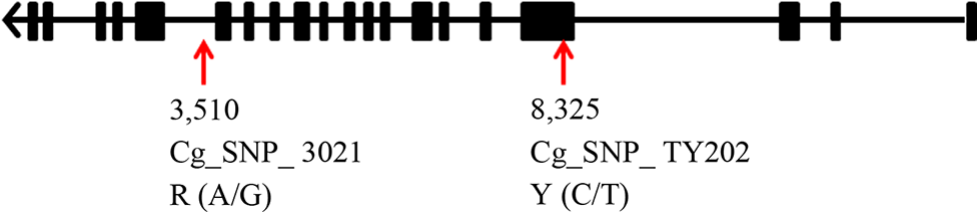
Location of the two associated SNPs in *Cg_GD1*.

Through the first stage of candidate gene association analysis with the 86 SNPs genotyped by HRM, we got one SNP (Cg_SNP_TY202, Y (C/T)) associated with glycogen content (P<0.01). It was located in the third exon of *Cg_GD1*. It was a synonymous mutation, and did not change the amino acid composition. The location and gene information of the SNP is shown in Table 3 and Fig 1. In the JN population, individuals with the TT and TC genotypes had much higher glycogen contents than those with the CC genotype (P<0.05). Individuals with the CC genotype were observed at a much lower frequency in the STG population (0.007) than in the JN population (0.11). We only found one individual with the CC genotype among the 144 individuals from the STG population (S1 Table). In addition, the average glycogen content of STG individuals was significantly higher at 6.17mg/g (P<0.01) than in JN individuals (5.35 mg/g) (S8 Table). This might support the finding that TT and TC individuals from the JN population had much higher glycogen contents than those with the CC genotype.

**Table 3:** Information for all the SNPs associated with glycogen content.

SNPs	Scaffold	Position	Allels	P.value	Enzyme	Area	Genotyping method
Cg_SNP_TY202	41994	8325	C/T	0.0056	*Cg_GD1*	exon	HRM
Cg_SNP_3021	41994	3510	A/G	0.0058	*Cg_GD1*	intron	sequencing
Cg_SNP_4	1083	167235	C/T	0.0064	*Cg_GP1*	up stream	sequencing

Area was the relative location of the SNP to the gene. *Cg_GD1*, glycogen debranching enzyme encoding gene 1. *Cg_GP1*, glycogen phosphorylase encoding gene 1.

**Fig 2:**
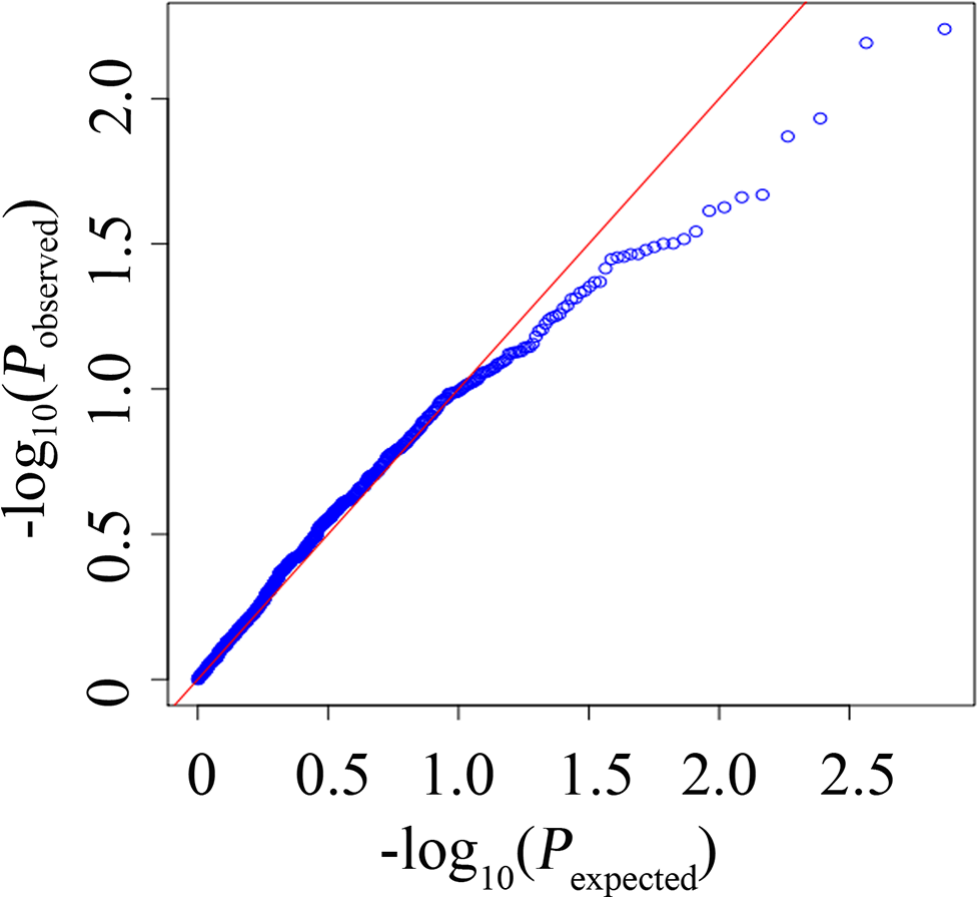
Quantile-quantile plot for glycogen content.

**Fig 3:**
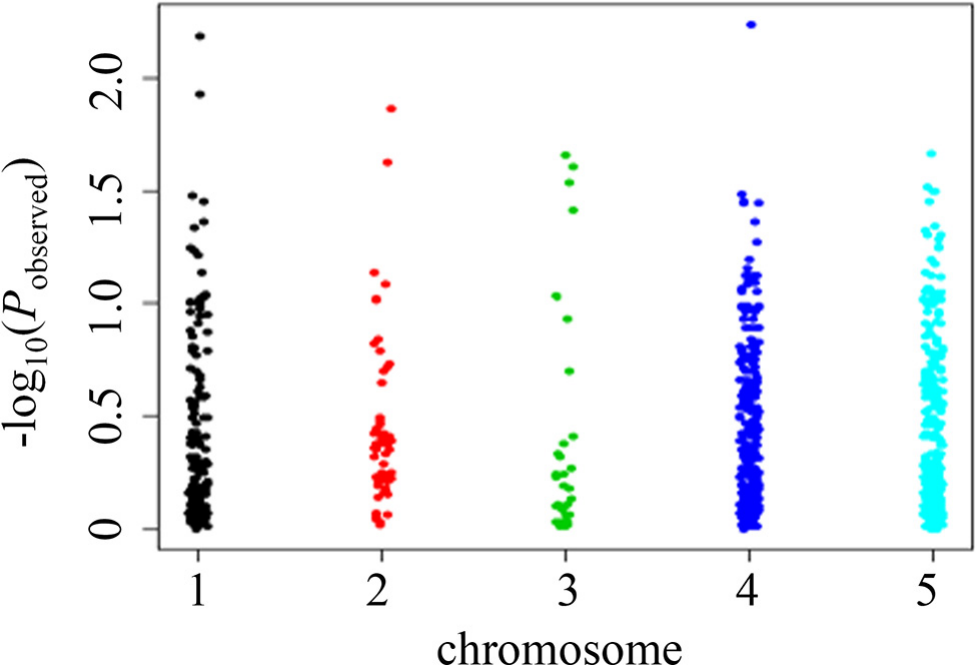
A Manhattan plot showing the associations of all SNPs.

We used the 732 high quality SNPs obtained via sequencing for the second stage of our association analysis. Two of the five targeted genes were located on two different linkage groups (unpublished data), and the other three were not mapped. We assumed that the different targeted genes are located on different chromosomes (S9 Table), for convenience of analysis and display. A quantile-quantile plot (Fig 2) and a Manhattan plot of the p-value (Fig 3) were produced. The association analysis results showed that two SNPs (P<0.01) were associated with the target trait glycogen content. One was Cg_SNP_3021 (R (A/G)), located on the 15th intron of *Cg_GD1*. Cg_SNP_3021 was 4815 bp from Cg_SNP_TY202, which further validated that *Cg_GD1* was associated with glycogen content. Cg_SNP_TY202 failed to be located through genotyping by sequencing. According to the results of the HRM analysis, 54 of the 64 (S1 Table) sequenced oysters were homozygous at the locus Cg_SNP_TY202, and only 11 samples had allele C. We analyzed the sequencing reads of the 11 individuals with allele C at Cg_SNP_TY202, and found that the depth was either too high or too low for SNP calling. The other SNP (Cg_SNP_4, Y (C/T)) associated with glycogen content was located upstream of *Cg_GP1*, 2773 bp from 5'UTR. Table 3 and Fig 1 provide detailed information for all associated SNPs.

We then performed haplotype analysis. For *Cg_GD1*, three haplotypes were established by PHASE using Cg_SNP_3021 and Cg_SNP_TY202 (S10 Table). The haplotypes were AT, AC, and GT, and the frequencies were 0.90, 0.08, and 0.02 respectively. Because of the uneven distribution of the allele frequency and the lack of haplotype GC individuals, there was no clear pattern of glycogen content across different haplotypes.

### The mRNA expression of associated genes

**Fig 4:**
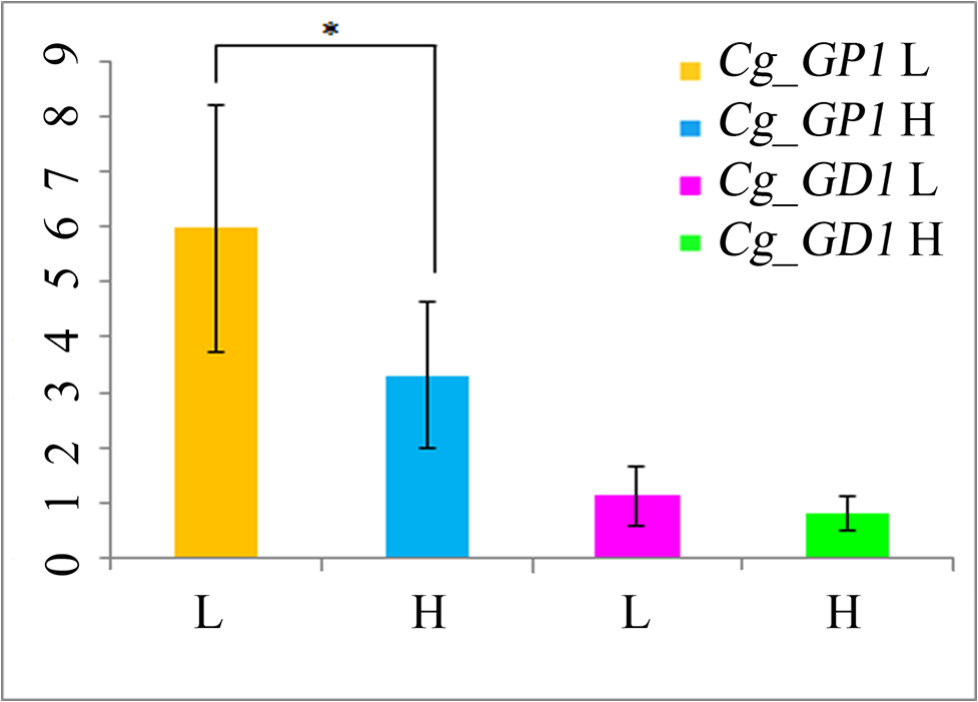
The mRNA expression levels of the high glycogen content and the low glycogen content groups. * P < 0.05, the difference between the high glycogen content and the low glycogen content group of *Cg_GD1* was significant. *Cg_GD1*, glycogen debranching enzyme encoding gene 1. *Cg_GP1*, glycogen phosphorylase encoding gene 1.

The glycogen content of the high- and low-glycogen content groups was 9.39 ± 0.48 (mg/g) and 3.15 ± 0.62 (mg/g), respectively. For *Cg_GP1*, the transcript abundance in the high glycogen content group was significantly down-regulated, compared that of the low glycogen content group (P<0.05). For *Cg_GD1*, the expression difference between the two groups was not significant (P>0.05), but the transcript abundance in the high glycogen content group was almost 50% lower than that of the low group (Fig 4). The lack of significant differences in *Cg_GD1* expression might due to the presence of multiple mutations in the genes determining glycogen content. In addition, the identified loci could only explain a part of phenotypic variation, and thus did not govern the consistency of *Cg_GD1* expression within groups. This could lead to differential expression within groups and a lack of significant differences between groups. Whether significant or not, the expression differences between the two genes implied that it is possible that polymorphisms associated with the Cg_SNP_TY202, Cg_SNP_3021, and Cg_SNP_4 might regulate the expression of *Cg_GP1* and *Cg_GD1* [38]. These data support the results of the association analysis, which indicated that *Cg_GP1* and *Cg_GD1* are associated with glycogen content at the transcript level.

The two enzymes both participated in the catabolism of glycogen, suggesting that it might be catabolism rather than anabolism that decides an individual’s glycogen content. It has been reported that triploid oysters had higher glycogen contents and lower physiological activity levels than diploid oysters; the mortality rate of triploids was about half that of diploids, and triploids produced higher quality meat than diploids [1,2]. It was deduced that oysters with higher glycogen contents, which did not exhibit active glycogen catabolism, performed better with respect to environmental stress resistance and nutritional status. The identified SNPs located on *Cg_GP1* and *Cg_GD1*, which participate glycogen catabolism, are potentially useful for the molecular breeding of the oysters.

## Conclusions

From the glycogen metabolism pathway of oysters, we screened 90 encoding genes of eight enzymes. Two hundred and ninety-five SNPs were validated via HRM analysis; 732 SNPs were called by resequencing the candidate genes and the 3 kb upstream and downstream regions of the genes. The candidate glycogen content genes and SNPs are valuable genetic resources for further studies on oyster. Cg_SNP_TY202 and Cg_SNP_3021 in *Cg_GD1*, and Cg_SNP_4 in *Cg_GP1* are associated with the glycogen content. The transcript abundance differences between the low and the high glycogen content individuals suggest that it is possible that the transcript regulation of *Cg_GD1* and *Cg_GP1* is mediated via the variations associated with associated SNPs. These findings will not only provide insights into the genetic basis of oyster quality traits, but also promote the research on the molecular breeding of oysters.

## Supporting Information

S1 Fig Histogram of glycogen content. (TIF)

S2 Fig The glycogen metabolism pathway. Yellow indicates enzymes that we selected as candidate genes. White indicates the substrates of those enzymes. UDPG is uridine diphosphate glucose. Glycogen G (n+1) unbranched contains Alpha1, 4-glycosidic bond only. Glycogen G (n+1) contains both Alpha1, 4-glycosidic bond and Alpha1, 6-glycosidic bond.
(TIF)

S1 Table Statistics of samples used in the experiment. 64 included 32 with the highest glycogen content and 32 with the lowest from the original 144 STG individuals. 32 included 16 with the highest glycogen content and 16 with the lowest from the original 144 STG individuals.
(XLSX)

S2 Table Operation table for glycogen content assay. The 0.01 mg/mL standard solution was diluted from the 1 mg/mL standard reserve liquid. The color reagent was made from color powder and concentrated sulfuric acid.
(XLSX)

S3 Table List of candidate genes for the glycogen metabolism pathway. (XLSX)

S4 Table Information for the 295 validated SNPs. Gene was the gene ID in which SNP located. CDS, coding sequence. INT, intron. INTER, intergenic region.
(XLSX)

S5 Table Information for genes selected for targeted gene resequencing. (XLSX)

S6 Table Details for the remaining 42 pairs of primers for targeted gene resequencing. (XLSX)

S7 Table Descriptive statistics for the low and high glycogen content groups. (XLSX)

S8 Table The descriptive statistics of different genotypes for Cg_SNP_TY202 in the two populations. The unit is mg/g.
(XLSX)

S9 Table The putative chromosome number for each gene. (XLSX)

S10 Table The descriptive statistics of different haplotypes. (XLSX)
