# Supplementary figures and images for: Candidate Gene Polymorphisms and their Association with Glycogen Content in the Pacific Oyster Crassostrea gigas
Source: PLoS One. 2015 May 7;10(5):e0124401. doi: 10.1371/journal.pone.0124401 (PMC4423957; doi:10.1371/journal.pone.0124401)

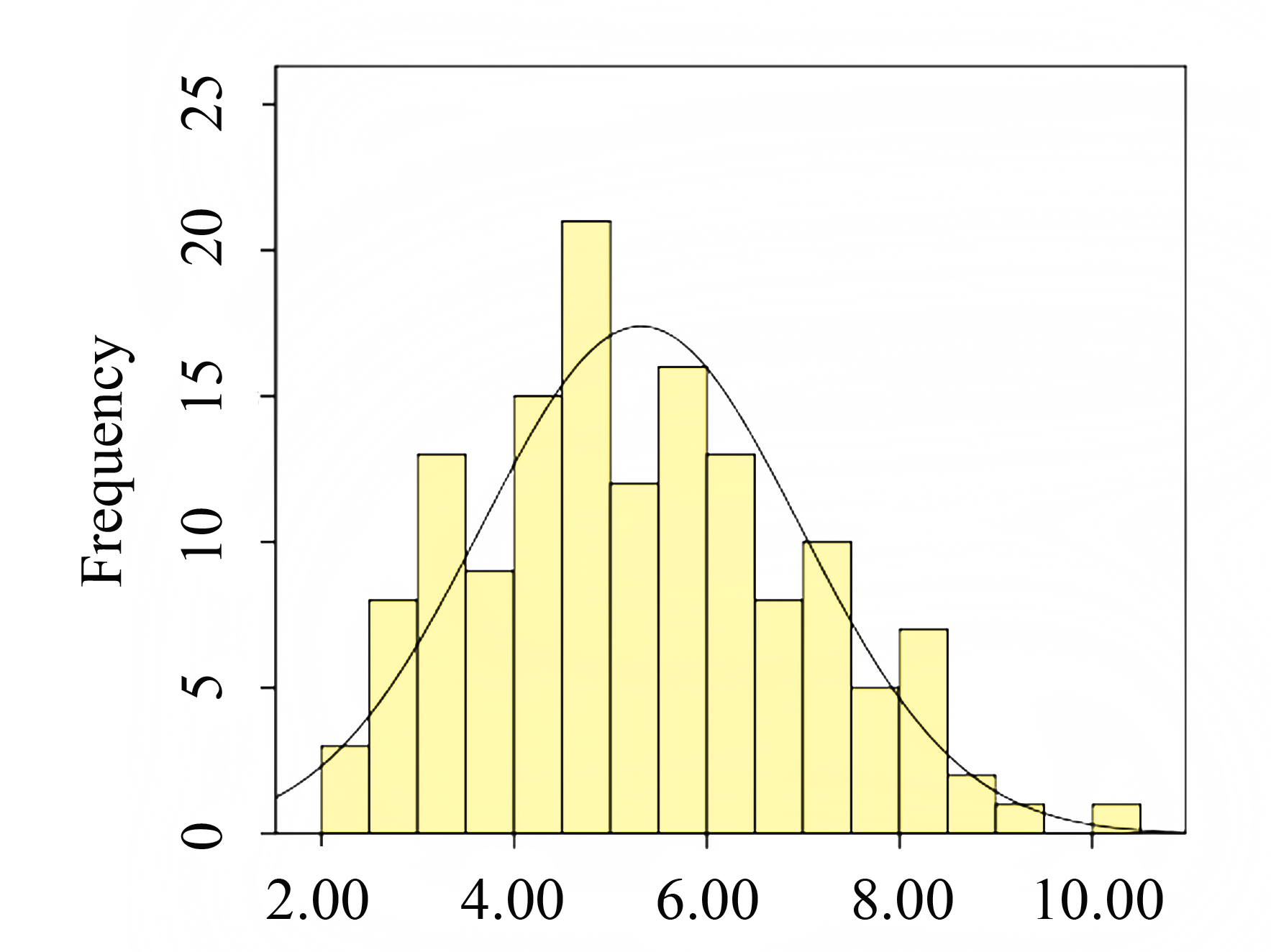

Supplement: S1 Fig — (TIF) [file pone.0124401.s001.tif]

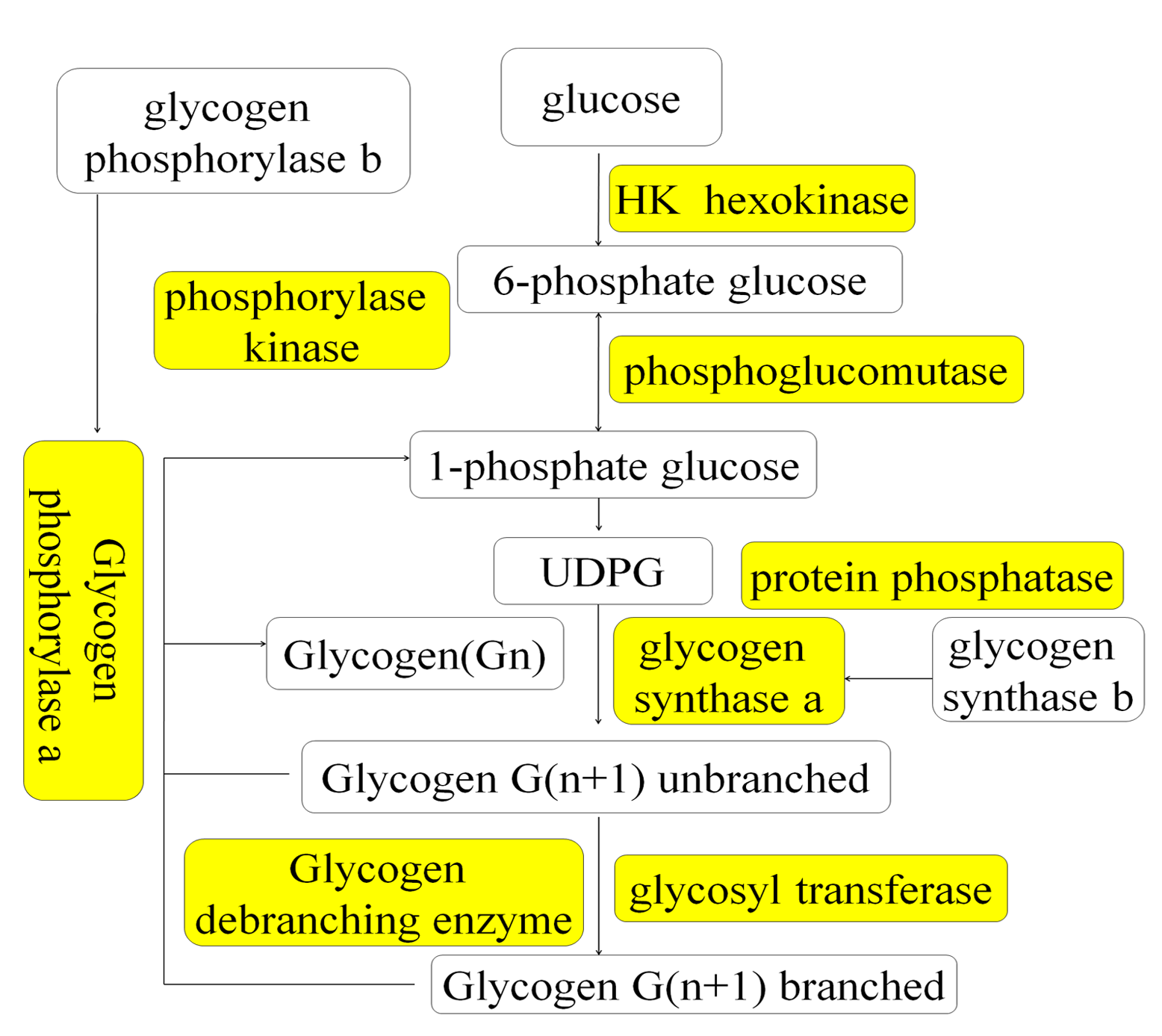

Supplement: S2 Fig — Yellow indicates enzymes that we selected as candidate genes. White indicates the substrates of those enzymes. UDPG is uridine diphosphate glucose. Glycogen G (n+1) unbranched contains Alpha1, 4-glycosidic bond only. Glycogen G (n+1) contains both Alpha1, 4-glycosidic bond and Alpha1, 6-glycosidic bond. (TIF) [file pone.0124401.s002.tif]
